# Strength and Fracture Mechanism of an Ultrafine-Grained Austenitic Steel for Medical Applications

**DOI:** 10.3390/ma14247739

**Published:** 2021-12-15

**Authors:** Gennadiy V. Klevtsov, Ruslan Z. Valiev, Natal’ya A. Klevtsova, Maxim N. Tyurkov, Mikhail L. Linderov, Marina M. Abramova, Arseniy G. Raab, Timur B. Minasov

**Affiliations:** 1 Department of Nanotechnology, Materials Science, and Mechanics, Togliatti State University, 445020 Togliatti, Russia; klevtsov11948@mail.ru (G.V.K.); inshtet@mail.ru (N.A.K.); turkovmn@mail.ru (M.N.T.); 2 Institute of Physics of Advanced Materials, Ufa State Aviation Technical University, 450008 Ufa, Russia; abramova.mm@ugatu.su (M.M.A.); agraab@mail.ru (A.G.R.); 3 Research Institute of Progressive Technologies, Togliatti State University, 445020 Togliatti, Russia; dartvi@gmail.com; 4 Department of Traumatology and Orthopedics, Bashkir State Medical University, 450008 Ufa, Russia; m004@ya.ru

**Keywords:** severe plastic deformation, equal-channel angular pressing, ultrafine-grained materials, austenitic steel, microstructure, mechanical properties, strength, fracture

## Abstract

In this paper, we study the corrosion-resistant austenitic steel Fe-0.02C-18Cr-8Ni for medical applications. The microstructure and mechanical properties (tensile mechanical properties, torsional strength, impact toughness, and static and cyclic crack resistance) under different types of loading of the steel are investigated. The results are compared for the two states of the steel: the initial (coarse-grained) state and the ultrafine-grained state produced by severe plastic deformation processing via equal-channel angular pressing. It is demonstrated that the ultrafine-grained steel 0.08C-18Cr-9Ni has essentially better properties and is very promising for the manufacture of medical products for various applications that experience various static and cyclic loads during operation.

## 1. Introduction

A wide use of “preserving” surgical technologies that enable minimizing traumatization during surgery and reducing the time of post-surgical rehabilitation in traumatology and maxillofacial surgery, involves miniaturization of medical products; for example, various implants, plates for bone osteosynthesis, and pins and screws for the fixation of plates and bone fragments. During operation the products may experience different stresses, both in terms of value and loading type: static and cyclic [1]. Therefore, the task of miniaturization of medical products cannot be solved without the use of materials having a high biocompatibility and a high set of mechanical properties under different types of loading [2,3,4]. These requirements are fully satisfied by a new class of bulk nanostructured metallic materials with an ultrafine-grained (UFG) structure produced by severe plastic deformation (SPD) processing [5,6,7]. Numerous studies provide convincing evidence that the UFG structure formation considerably increases the hardness, strength, and fatigue life of materials, which will enable miniaturizing products used in medicine [5,6,7,8]. For example, the testing of plates for medical applications in maxillofacial surgery, produced from SPD-processed nanostructured Ti, showed that the mechanical properties and fatigue endurance of such plates were higher by a factor of almost 1.5 as compared to plates from conventional coarse-grained Ti [2].

The same approach is fully applicable to corrosion-resistant austenitic steels widely used in traumatology and other areas of medicine [9,10,11]. Therefore, the effect of the UFG structure on the fracture resistance of austenitic steels under different types of loading remains an issue of current importance.

The aim of the present study is to evaluate the strength and fracture mechanisms under different types of loading of a UFG austenitic steel for medical applications in comparison with its coarse-grained (CG) counterpart.

## 2. Materials and Methods

We selected the corrosion-resistant austenitic steel Fe-0.02C-18Cr-8Ni (see Table 1 for the compete chemical composition), widely used in medicine, as the material to be studied in the UFG and CG states.

The austenitic steel Fe-0.02C-18Cr-8Ni was investigated in the initial CG state, produced by quenching from a temperature of 1050 °C with a preliminary holding, and in the UFG state. The UFG state of the billets was produced by equal-channel angular pressing (ECAP) [5,7,11], using a die-set with channels 20 mm in diameter intersecting at an angle of φ = 120°, at the die-set temperature of 350 °C. The billets were heated separately in a furnace to a temperature of 350 °C, and held under the condition of billet heating to a section diameter of 1.5 mm per minute prior to each processing cycle. The billets were processed via route Bc, where a billet is rotated 90° with respect to its longitudinal axis after each cycle, for 4 deformation cycles (1 ECAP pass at an angle of 120° corresponds to an equivalent strain of ~0.7–0.8). The pressing rate was about 6 mm/s. After the ECAP processing, the billets of the material under study had a diameter of about 20 mm and a length of about 100 mm.

The CG structure was studied using metallographic microscopes GX50 (Olympus, Tokyo, Japan) and Axiovert 40 MAT (Carl Zeiss, Oberkochen, Germany). Sandpaper was applied to metallographic specimens; its grit was increased each time, eventually to 4000, and the grinding direction was changed 90°. To reveal grain boundaries, a supersaturated solution of oxalic acid was used. The mean grain size was determined by the linear intercept method [12], measuring at least 300 grains. The fine structure of the UFG steel was studied using a JEM-2100 transmission electron microscope (TEM) (JEOL, Tokyo, Japan), and the accelerating voltage was 200 kV. To prepare foils, a 10% solution of perchloric acid in butanol was used. The voltage was 45–50 V. The hardness tests were performed using a TH 300 hardness tester (Beijing TIME High Technology Ltd., Beijing, China). The loading time was 3 s and the exposure time was 2 s. The static tension of the cylindrical specimens with a diameter of 3 mm was carried out at temperatures of 20 and −196 °C (a test in a liquid nitrogen medium is required to evaluate the static crack resistance of a steel under plane strain (PS) [13,14]) using an H50KT universal testing machine (Tinius Olsen, Redhill, UK). The grip movement rate was 5 mm/min. The impact toughness tests (KCV) of the specimens 10 × 10 × 55 mm in size with a V-shaped stress raiser were performed using a JB-W300 computer-controlled pendulum impact testing machine (TIME Group Inc., Beijing, China). The fatigue tests and the static crack resistance tests were conducted on prismatic specimens 10 mm in thickness, 15 mm in height, and 80 mm in length via three-point bending using an Instron 8802 testing system (High Wycombe, UK). The fatigue tests were carried out at a temperature of 20 °C with a loading frequency of ⱱ = 10 Hz, a loading ratio of R = 0.1, and different values of load (ΔP). The static crack resistance tests were performed, in compliance with the Russian standard GOST 25.506-85 [14], at temperatures of 20 and −196 °C. In the latter case, the specimens were tested in a liquid nitrogen medium using a specially made Dewar flask. The torsion tests of the cylindrical specimens with a gauge diameter of 10 mm and a length of 100 mm were carried out at a temperature of 20 °C using an MK-50 testing system (Moscow Experimental Plant of Testing Machines and Balances, Moscow, Russia) in compliance with the Russian standard GOST 3565-80, taking into account the standard GOST R 50581-93 (ISO 6475-89). The mechanical properties of the steel under torsion were calculated from the “torque—angle of twist” diagram. The microfractographic studies of the fracture surfaces were performed using a JCM-6000 scanning electron microscope (SEM) (JEOL, Tokyo, Japan).

## 3. Results

### 3.1. Microstructure and Tensile Mechanical Properties of the Steel

**Figure 1:**
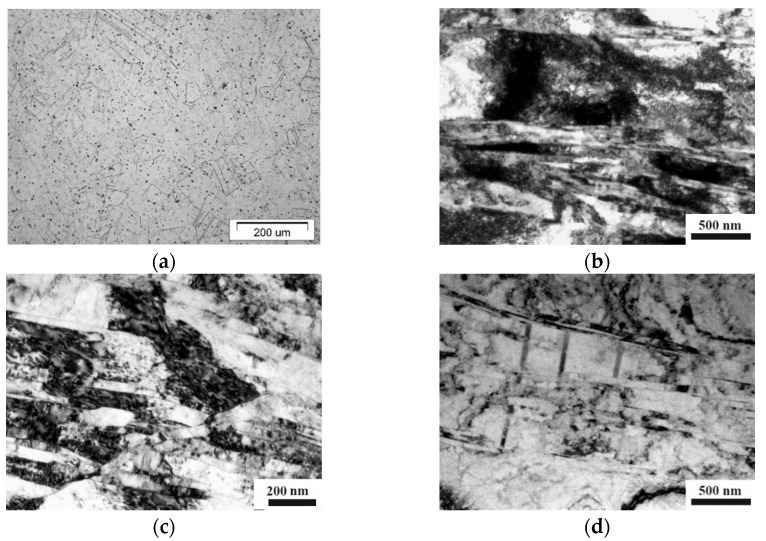
Microstructure of the austenitic steel in the initial state, optical microscopy (**a**) and ECAP-processed state, TEM (**b**–**d**).

In the initial state, the structure represented equiaxed austenitic grains with a diameter of about 30 μm. Additionally, multiple annealing twins were observed in the structure (Figure 1a).

In the ECAP-processed steel Fe-0.02C-18Cr-8Ni, an elongated banded UFG structure is observed (Figure 1b–d). Against the background of a developing cellular structure, shear microbands and bands are formed (Figure 1b,c). Shear bands with a thickness of up to 100 nm are formed inside distinct mesobands with a thickness reaching 700 nm. In addition, both annealing twins and deformation twins are observed in the structure (Figure 1d). In the interior of the mesobands, a high dislocation density, dislocation pile-ups and coils are present (Figure 1b). The mean grain size and tensile mechanical properties of the steel are presented in Table 2.

Thus, the steel in the initial state has a rather low hardness and strength and a high ductility. After ECAP processing, the hardness and tensile strength of the steel increase by a factor of 1.8–3.8, whereas the ductility decreases by a factor of 4.

### 3.2. Static Crack Resistance of the Steel

Since medical products have different sizes and configurations, there may be different local stress states at the tip of a crack that emerges [13,14,15]. Therefore, it is necessary to have information about the static crack resistance (K_1C_) [13] of the steel under plane strain and about the static crack resistance (K_C_) where the PS conditions are not realized.

It was impossible to determine the static crack resistance of the CG steel Fe-0.02C-18Cr-8Ni at 20 °C due to its high ductility, and a decrease in the test temperature to −196 °C did not lead to the realization of the PS conditions (Table 3). The test results of the UFG steel specimens at temperatures of 20 and −196 °C demonstrate that the steel in the UFG state has a high crack resistance (above 90 MPa√m). However, none of fracture mechanics criteria satisfy the PS condition (Table 3) [13,14]. Consequently, the static crack resistance values for the CG and UFG steel Fe-0.02C-18Cr-8Ni obtained at test temperatures of 20 and −196 °C represent K_C_ [13].

**Figure 2:**
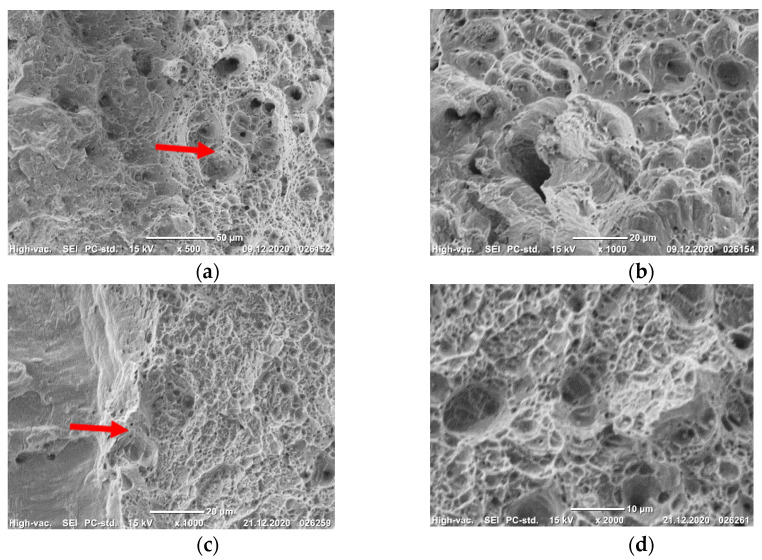

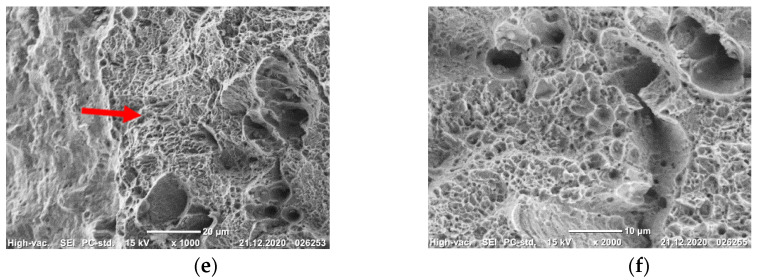
Fracture surface microrelief at the fracture nucleus (**a**,**c**,**e**) and in the static fracture region (**b**,**d**,**f**) of the CG (**c**,**d**), and UFG (**a**,**b**,**e**,**f**) specimens from the steel tested for static crack resistance at 20 °C (**a**,**b**) and −196 °C (**c**–**f**). The arrows indicate the nucleus of static fracture. (**a**)—×500; (**b**,**c**,**e**)—×1000; (**d**,**f**)—×2000.

The steel in the CG and UFG states at temperatures of 20 and −196 °C fractures in a ductile manner with the formation of a dimple microrelief (Figure 2a–f). This is one of the reasons why the condition of plane strain was not achieved even at a temperature of −196 °C [15].

Thus, the steel Fe-0.02C-18Cr-8Ni in the UFG state has a higher static crack resistance K_c_ (above 90 MPa√m) as compared to its CG counterpart. Irrespective of its state, the steel fractures in a ductile manner with the formation of a dimple microrelief down to a temperature of −196 °C.

### 3.3. Torsional Strength of the Steel

Since many medical products, e.g., screws, experience torsional loads, it is interesting to evaluate the resistance to torsion of the UFG steel Fe-0.02C-18Cr-8Ni in comparison with its CG counterpart.

**Figure 3:**
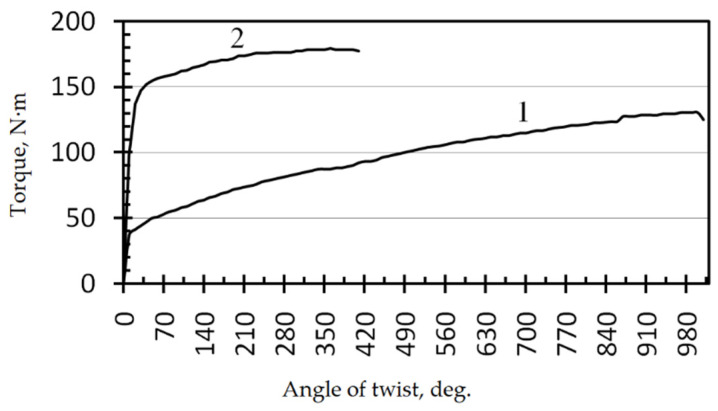
“Torque—angle of twist” diagram based on the torsion tests of the specimens from the CG (**1**) and UFG (**2**) steel.

The torsion tests of the steel specimens show (Figure 3) that the torque, corresponding to macroscopic yielding, of the UFG steel is higher than that of the CG steel. The number of revolutions and the angle of twist of the UFG steel specimens are lower than those of the CG steel specimens (Table 4). The ultimate torsional strength and torsional yield strength of the UFG steel increase by factors of 1.3 and 3.8, respectively, as compared with the CG steel, while the relative shear decreases by a factor of 2.4.

Thus, it can be seen that the steel in the UFG state has a better resistance to torsional fracture than the steel in the CG state.

**Figure 4:**
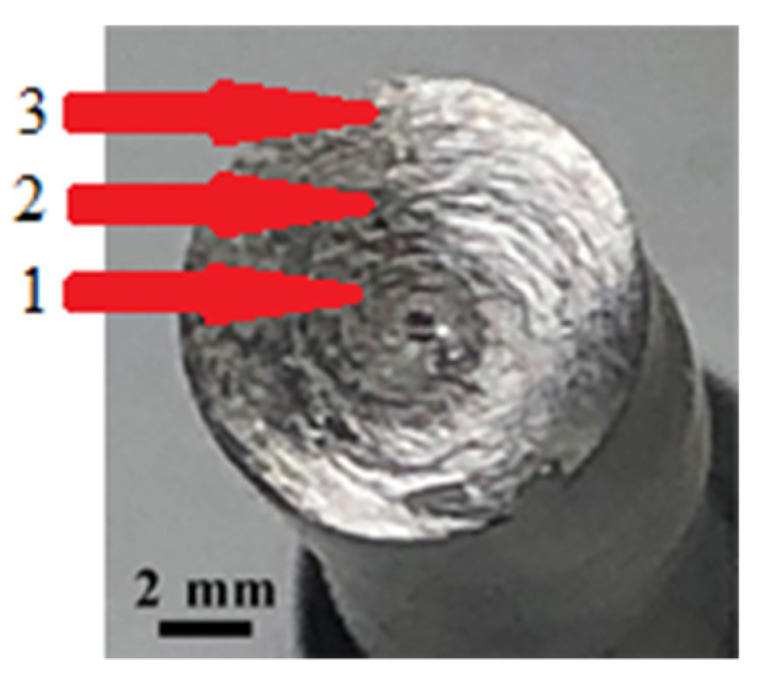
Typical view of the fracture surface of the steel specimen during torsion: (**1**) central part, (**2**) transition (middle) part, and (**3**) peripheral part.

**Figure 5:**
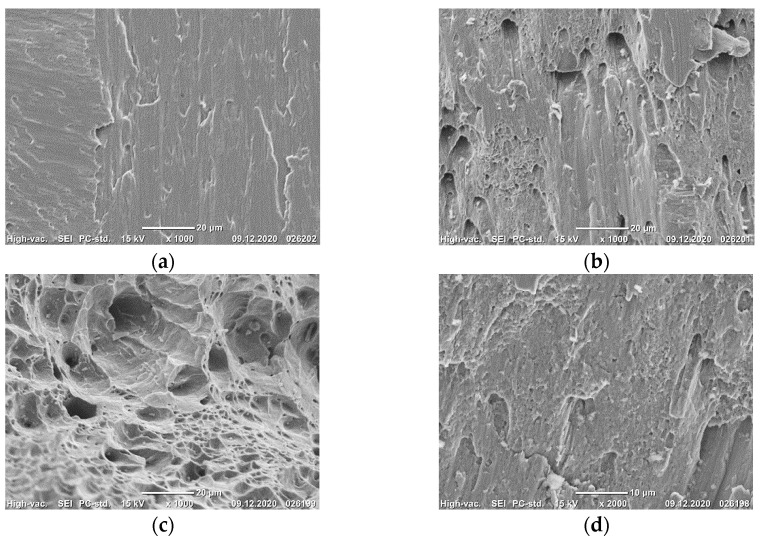

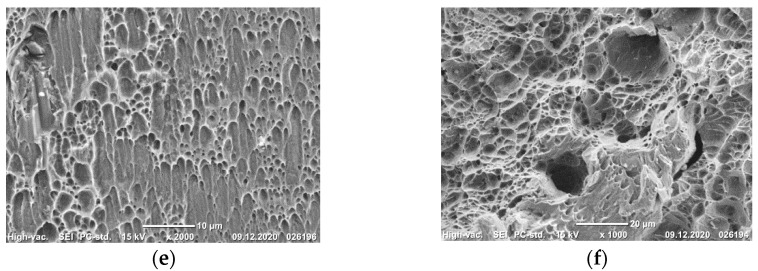
Torsional fracture surface microrelief of the specimens from the CG (**a**–**c**) and UFG (**d**–**f**) steel. The microrelief was taken from the peripheral (**a**,**d**), middle (**b**,**e**), and central (**c**,**f**) parts of the fracture surface. (**a,b,c,f**)—×1000; (**d,e**)—×2000.

Three regions can be distinguished in all the fracture surfaces: the ductile central part, the transition (middle) part, and the relatively smooth peripheral part (Figure 4). The fracture surface microrelief reflects the process of specimen fracture during torsion. Fracture under torsional stresses starts with the formation of shear dimples in the peripheral and middle regions of the fracture. During the further torsion of the specimen, the formed shear dimples turn out to be fully rubbed out in the peripheral region (Figure 5a,d) as a result of a mutual friction between the mating fracture surfaces. In the middle region of the fractures of the CG steel, the shear dimples are heavily rubbed out (Figure 5b); in the fracture of the UFG steel, likely due to the higher hardness of the steel, the shear dimples are preserved and alternate with the rubbed-out surface areas (Figure 5e). In the central part, fracture occurred under normal rupture stresses as evidenced by the predominantly equiaxed dimples (Figure 5c,f).

### 3.4. Impact Toughness of the Steel

The impact toughness (KCV) tests of the specimens from the CG and UFG steel Fe-0.02C-18Cr-8Ni demonstrate that the KCV of the CG steel is visibly higher than that of the UFG steel (Table 5). Such a difference in the impact toughness values is apparently conditioned by certain features of the impact crack initiation and propagation in CG and UFG steels. To gain an understanding of this phenomenon, let us consider the impact fracture mechanism of the steel.

**Figure 6:**
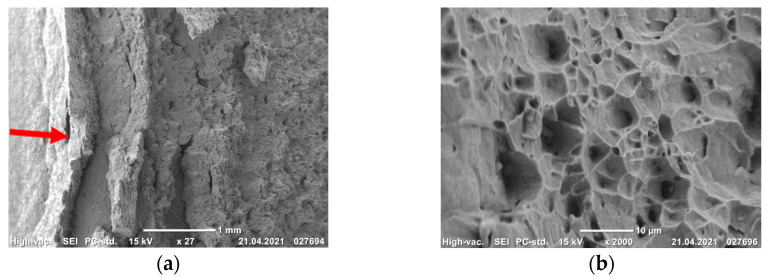

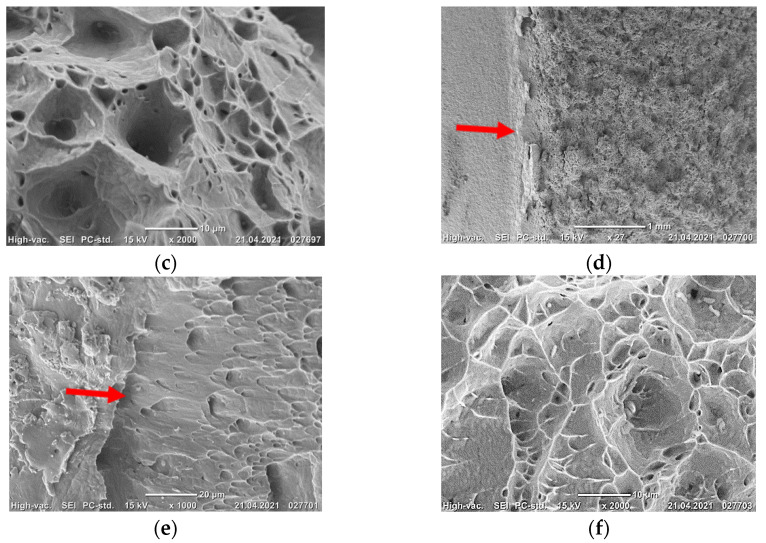
Microrelief of the impact fracture surfaces of the CG (**a**–**c**) and UFG (**d**–**f**) steel in the crack initiation nucleus (**a**,**b**,**d**,**e**) (nucleus marked by an arrow) and in the central part of the fractures (**c**,**f**). (**a**,**d**)—×30; (**b**,**c**,**f**)—×2000; (**e**)—×1000.

All of the produced fractures of the steel Fe-0.02C-18Cr-8Ni, irrespective of the steel’s state, had a fibrous structure and shear lips [15,16]. On the impact fracture surfaces of the CG steel in the crack initiation nucleus, we observe an L zone with a length of about 2 mm, described in [17]. The L zone has a considerable roughness in the form of ductile ridges located in parallel to each other (Figure 6a). It can be seen at a large magnification (Figure 6b) that the microrelief of this zone consists of deep and smooth equiaxed dimples. The L zone formation indicates a high value of the crack initiation work under the impact loading of the specimens [17]. In the central part of the fractures, the microrelief also consists of deep and smooth equiaxed rupture dimples with different sizes (Figure 6c), indicating a high energy capacity of fracture.

On the fracture surface of the UFG steel, the *L* zone is absent in the crack initiation nucleus (Figure 6d). At a large magnification, a smooth draft microzone ϴ [16] with a length of 50–60 μm can be seen in the crack initiation nucleus. The microrelief of this zone consists of shallow shear dimples (Figure 6e). In the central part of the fracture, shallow rupture dimples of different sizes are observed (Figure 6f). Such a fracture surface microrelief of the UFG steel specimens is evidence of a low energy capacity of fracture as compared to the CG steel. 

### 3.5. Fracture Kinetics and Mechanism of the Steel in the Low-Cycle Fatigue Region

It is known that the majority of failures of medical implants occur in the low-cycle fatigue region [1]. At present, to analyze a material’s resistance to fatigue fracture in the low-cycle fatigue region, kinetic diagrams of fatigue fracture are used that describe the dependence of the fatigue crack propagation rate on the stress intensity coefficients ΔK or K_max_ [18].

**Figure 7:**
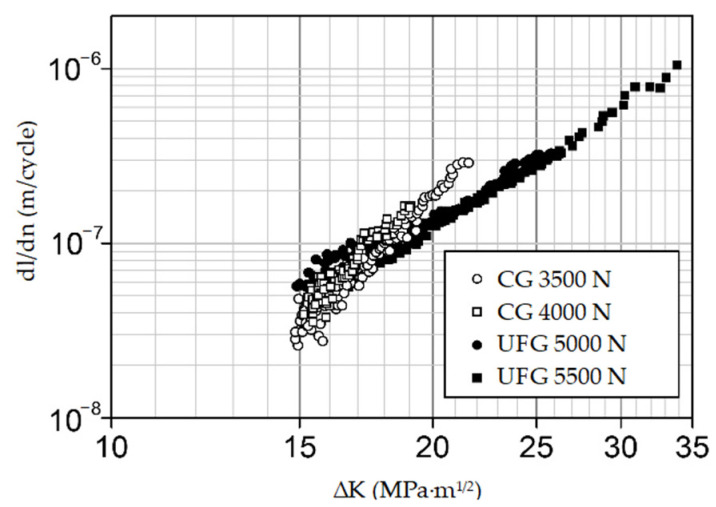
Straight-line portion of the kinetic diagrams of fatigue fracture for the steel in the initial state (bright dots) and after ECAP (dark dots).

Analysis of the kinetic diagrams of fatigue fracture for the steel shows (Figure 7) that at the same value of the coefficient ΔK, the fatigue crack propagation rates in the CG and UFG steels differ insignificantly, especially at the low values of ΔK. However, as it can be seen from Table 6, the coefficient n in the Paris equation [19] for the UFG steel is lower than that for the CG steel (3.5 in contrast to 6.0). Consequently, the steel in the UFG state is less sensitive to cyclic loads emerging during product operation as compared to the CG steel [15,18].

**Figure 8:**
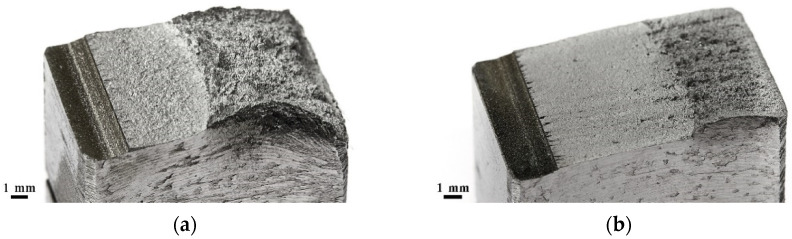
General view of the fatigue fractures of the specimens from the CG (**a**) and UFG (**b**) steel.

On the surfaces of all the fatigue fractures of the steel Fe-0.02C-18Cr-8Ni, two zones are visible: the smooth zone of a crack’s fatigue propagation, l_f_, and the fibrous zone of final failure (Figure 8a,b) [15]. A high value of the coefficient ΔK at the boundary of the l_f_ zone during the fracture of the UFG steel (Figure 7) and, consequently, a large length of the l_f_ zone in comparison to the CG steel (Figure 8) indicate [15,18] that the UFG steel has a higher cyclic crack resistance than the CG steel.

**Figure 9:**
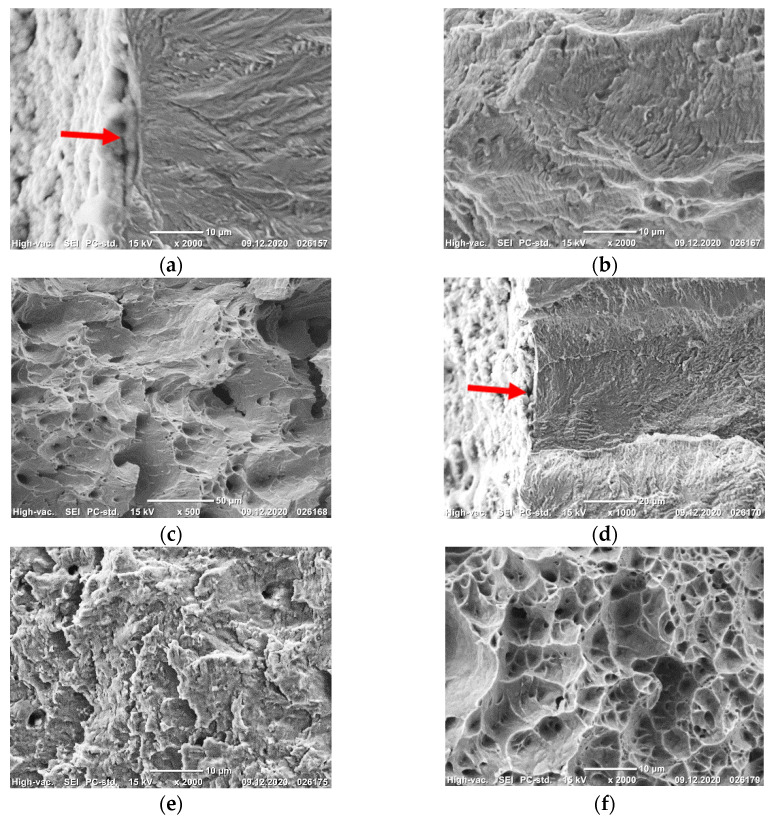
Microrelief of the fatigue fracture surface of the CG (**a**–**c**) and UFG (**d**–**f**) steel in the vicinity of the fracture nucleus (**a**,**d**), in the middle area of the crack length (**b**,**e**) and in the final failure zone (**c**,**f**). The fatigue fracture nucleus is indicated by arrows. The direction of crack propagation is from left to right. (**a**,**b**,**e**,**f**)—×2000; (**c**)—×500; (**d**)—×1000.

The fatigue fractures of the CG steel in the vicinity of the crack initiation nucleus have a microrelief oriented in the direction of the fatigue crack propagation (Figure 9a). Meanwhile, in the fractures of the UFG steel, ductile fatigue striations can be observed already in the vicinity of the fracture nucleus (Figure 9d). In the fractures of the CG steel, in the middle area of the l_f_ zone, ductile fatigue striations and secondary cracks parallel to them are visible (Figure 9b). In the fractures of the UFG steel, secondary cracks are more numerous and larger in size (Figure 9e). Closer to the final failure, the number of cracks on the fracture surfaces of the CG and UFG steels increases. Irrespective of the steel state, the final failure zone has a dimple structure with deep, smooth dimples (Figure 9c,f).

Thus, the research results demonstrate that the steel Fe-0.02C-18Cr-8Ni in the UFG state has a higher cyclic crack resistance and is less sensitive to cyclic overloads.

## 4. Discussion

As noted above, in traumatology and other areas of medicine, corrosion-resistant austenitic steels are widely used for the production of various implants (plates, screws, pins, etc.) as well as tools and accessories for their installation. In the process of operation, medical products experience loads large in value and various in type. Therefore, when selecting a material for their production, it is not sufficient to have data only about the basic characteristics (hardness and tensile strength); it is necessary to also take into account a whole set of mechanical properties found under different types of loading. Hence, it is interesting to compare a set of mechanical properties of the corrosion-resistant austenitic steel Fe-0.02C-18Cr-8Ni, widely applied in medicine, in the ECAP-produced UFG state with the respective properties of the steel in the CG state.

As shown above, after ECAP processing, the steel’s hardness and tensile strength increase by a factor of 1.8–3.8, while ductility decreases by a factor of 4. However, the decline in the ductility of the steel in the UFG state did not lead to a decline in other important strength characteristics, such as static crack resistance, torsion resistance, and fatigue strength. An exception is impact toughness.

Let us consider the importance of the above-mentioned strength characteristics of the steel in terms of medical products.

Many medical products that experience large static loads during operation have a complex configuration with stress raisers, not excluding the presence of cracks caused, for instance, by implant deformation during their tailoring to meet the patient’s requirements. In this case, the capability of steel to restrain crack propagation will depend on the static crack resistance of the steel, taking into account the material’s local stress state at the crack tip: K_1C_ under plane strain (for relatively large products) or K_C_ under conditions where there is no PS [13,14] (for miniature implants). The performed tests show that the steel Fe-0.02C-18Cr-8Ni in the UFG state has a higher static crack resistance (K_c_ = 96.0 ± 1.02 MPa√m) than its CG counterpart (K_c_ = 60.8 ± 0.52 MPa√m). Consequently, it will better restrain the emerging cracks in products.

Analysis of the operational damages of medical products reveals [20,21] that a large percentage of fractures of screws for the fixation of plates and bone fragments in traumatology and other areas of medicine occurs by twisting in the smooth area of a screw between the threaded portion and the head. This happens most often during the unscrewing of screws fused with bone conducted after the recovery of a patient or due to other reasons [21]. The torsion tests of the samples demonstrate that the ultimate torsional strength and the torsional yield strength of the UFG steel increase by a factor of 1.3 and 3.8, respectively, while the relative shear decreases by a factor of 2.4, as compared to the CG steel. The decrease in the relative shear may also be a favorable factor since it increases the steel’s resistance to shear fracture.

Unlike one-time static loads, the UFG steel Fe-0.02C-18Cr-8Ni offers poor resistance to one-time impact loads. Impact toughness (KCV) tests show that the KCV of the CG steel is much higher than that of the UFG steel (2.9 vs. 0.7 MJ/m^2^).

It is known that ECAP processing increases the fatigue limit of most structural materials and has an ambiguous effect on fatigue strength in the low-cycle region [22,23]—the region where most of the fractures of medical implants take place [1]. The low-cycle fatigue tests of the samples show that the steel in the UFG state has a higher cyclic crack resistance and is less sensitive to cyclic overloads than its CG counterpart. 

Summarizing the above, the performed research demonstrates that the strength properties of the UFG steel Fe-0.02C-18Cr-8Ni in all types of static tests (tension, torsion, and static crack resistance), as well as in fatigue tests, are higher than those of the CG steel, except in impact toughness. Therefore, the steel in the UFG state is a more promising material, in comparison with the CG steel, for the manufacture of medical products for different applications that experience various static and cyclic loads during operation.

## 5. Conclusions

1. The equal-channel angular pressing of the austenitic steel Fe-0.02C-18Cr-8Ni for medical applications via the regimes described in this paper, through the formation of a UFG structure with a mean grain size of 0.55 μm and a high density of crystalline structure defects, noticeably increases the hardness and tensile strength properties of the steel and decreases the ductility.
2. The steel in the UFG state has a high static crack resistance K_c_ (above 90 MPa√m), but the impact toughness (KCV) of the UFG steel has declined, in comparison with the CG steel, from 2.9 to 0.7 MJ/m^2^. Irrespective of the steel state and the loading type, the steel fractures in a ductile manner with the formation of a dimple microrelief.
3. The ultimate torsional strength and the torsional yield strength of the UFG steel considerably increase, in comparison with the CG steel, which is a favorable factor reducing the probability of fracture during the unscrewing of screws fused with bone in bone osteosynthesis.
4. In comparison with the CG steel, the UFG steel has a higher crack resistance and, owing to the lower value of the coefficient n in the Paris equation (3.5 vs. 6.0), is less sensitive to cyclic overloads in the low-cycle fatigue region.
5. Thus, the UFG steel Fe-0.02C-18Cr-8Ni is a more promising material than its CG counterpart for the manufacture of medical products for various applications that experience various static and cyclic loads during operation. The use of the UFG steel could provide an opportunity for the miniaturization of products together with the preservation of the necessary strength characteristics that are presently required from a material.

## Figures and Tables

**Table 1:** Chemical composition analysis results for the austenitic steel under study, wt%.

C	Cr	Ni	Mn	Mo	Si	Cu	Co
0.023	17.95	7.95	1.85	0.35	0.38	0.6	0.15

**Table 2:** Mean grain size and tensile mechanical properties of the steel.

State	d_mean,_ μm	HB	σ_B_, MPa	σ_0.2_, MPa	δ, %
CG (initial)	30	159 ± 2	624 ± 6	283 ± 2	80 ± 0.7
UFG (ECAP)	0.55	363 ± 2	1112 ± 15	1065 ± 15	20 ± 0.5

**Table 3:** Static crack resistance of the steel in different states and the criteria for the PS conditions to be realized according to GOST 25.506-85.

Condition	T, °C	σ_0.2_, MPa	K_C_, MPa√m	t/(K_C_/σ_0.2_)^2^	P_max_/P_Q_	φ_c_, %
CG (initial)	−196	605 ± 23	60.8 ± 0.52	1.00	1.52	3.0
UFG (ECAP)	20	1065 ± 15	96.0 ± 1.02	1.24	1.20	4.0
UFG (ECAP)	−196	1282 ± 57	92.1 ± 1.68	1.95	1.05	1.5

**Table 4:** Mechanical properties of the steel.

State	Torque, N·m	Number of Revolutions, n	The Angle of Twist, Deg.	τ_k_, MPa	τ_0.3_, MPa	g, %
CG (initial)	133 ± 1.30	2.83 ± 0.014	1020 ± 5.0	688	194	89
UFG (ECAP)	181 ± 1.80	1.67 ± 0.019	420 ± 7.0	917	740	37

**Table 5:** Impact toughness (KCV) of the CG and UFG steel at a temperature of 20 °C.

State	KCV, MJ/m^2^
CG (initial)	2.9 ± 0.10
UFG (ECAP)	0.7 ± 0.15

**Table 6:** Paris equations describing the straight-line portion of the kinetic diagrams of fatigue fracture for the steel after different types of treatment.

CG (Initial)	UFG (ECAP)
$\frac{dl}{dN}=3.3\cdot 10^{-15}{\left(∆K\right)}^{6.0}$	$\frac{dl}{dN}=7.2\cdot 10^{-12}{\left(∆K\right)}^{3.5}$

## Data Availability

Not applicable.
